# 胸腔镜早期周围型肺癌根治术对患者术后炎性反应状态的影响

**DOI:** 10.3779/j.issn.1009-3419.2014.10.04

**Published:** 2014-10-20

**Authors:** 懿 刘, 洪林 赵, 京豪 刘, 毅 武, 嵩 徐, 高阳 林, 军 陈, 钢 陈, 清华 周

**Affiliations:** 300052 天津，天津医科大学总医院肺部肿瘤外科 Tianjin Medical University General Hospital of Lung Cancer Surgery, Tianjin 300052, China

**Keywords:** 胸腔镜, 肺肿瘤, 炎性反应, 肺叶切除术, Thoracoscopy, Lung neoplasms, Inflammatory reaction, Lobectomy

## Abstract

**背景与目的:**

胸腔镜治疗早期肺癌在预后方面取得与传统开胸手术方式相同的效果，患者在这两种术式术后恢复方面是否存在差别是最近研究的热点。本研究旨在探讨胸腔镜治疗早期周围型肺癌根治术患者机体术后炎性状态变化。

**方法:**

选取71例于天津医科大学总医院行早期周围型肺癌根治术的患者为研究对象，根据手术方式不同分为胸腔镜组35例和开胸手术组36例，比较两组患者手术时间、出血量、淋巴结清扫个数、术后胸管留置时间、胸引量及术前1 d，术后3 d和术后7 d的C-反应蛋白（C-reactive protein, CRP）、α-肿瘤坏死因子（tumor necrosis factor-α, TNF-α）、白介素-6（interleukin-6, IL-6）、IL-10的水平。

**结果:**

两组患者手术时间、出血量、淋巴结清扫个数、术后胸管留置时间、胸引量无明显差异，术后CRP、TNF-α、IL-6和IL-10的水平均较术前升高，胸腔镜组患者的升高幅度小于开胸手术组。

**结论:**

胸腔镜行早期周围型肺癌根治术对患者机体术后炎性反应状态影响更小。

## Abstract (en)

**Background and objective:**

It has been proven that video assited thoracoscopic surgery (VATS) achieved the same survival rates compared with traditional open chest operation in the treatment of early stage of lung cancer. but it is unclear if there is any difference of body inflammatory reaction between the two operation. The aim of this study is to investigate the changes of inflammatory state of thoracoscopic radical lobectomy in early peripheral lung cancer patients.

**Methods:**

Senventy-one early peripheral lung cancer patients who have underwent radical lobectomy were divided into two groups based on the different operation method. The VATS group was treated by thoracoscopic lobectomy. The thoracotomy group was treated by traditional thoracotomy. Then the level of serum C-reactive protein (CRP), tumor necrosis factor-α (TNF-α), interleukin-6 (IL-6) and IL-10 at 1-day before operation and 3-day, 7-day postoperation were measured and compared between the two groups.

**Results:**

No significant difference was found in the level of serum CRP, TNF-α, IL-6 and IL-10 before operation. Compared to the thoracotomy group, the level of serum CRP, TNF-α, IL-6 and IL-10 in the VATS group were significantly lower after operation.

**Conclusion:**

Compared with thoracotomy lobectomy, thoracoscopic lobectomy for early peripheral lung cancer patients is associated with lower inflammatory responses.

随着微创术式的不断发展，胸腔镜下早期肺癌根治术日臻完善，已成为治疗早期肺癌的基本微创术式。因其具有创伤小、恢复快等独特优势使得该术式后患者相关并发症明显降低。肺癌患者术后常见的并发症是肺部炎症，减少肺部炎症的发生，对手术后患者快速恢复有重要意义。观察肺部炎症的发生及严重程度有生化指标和临床指标两种，血清炎性因子与肺部及全身炎症反应程度密切相关，是手术后炎症反应程度的良好生化指标。本研究选取血清中肿瘤坏死因子-α（tumor necrosis factor-α, TNF-α）、C-反应蛋白（C-reactive protein, CRP）、白介素-6（interleukin-6, IL-6）及IL-10等炎性因子作为观察指标，以传统开胸手术作为对照，研究胸腔镜下早期肺癌根治术对患者炎性反应状态的影响。

## 1 资料与方法

### 1.1 一般资料

将2012年5月-2013年5月在天津医科大学总医院肺部肿瘤外科进行早期肺癌根治术治疗患者71例，按手术方式不同分为胸腔镜组和开胸手术组。胸腔镜组35例，男性19例，女性16例，年龄43岁-78岁，平均（62.7±4.9）岁，右下肺叶9例，右上肺叶7例，右中肺叶2例，左下肺叶9例，左上肺叶8例，术后病理鳞癌15例，腺癌19例，大细胞癌1例。开胸手术组36例，男性23例，女性13例，年龄41岁-75岁，平均（61.9±3.8）岁，右下肺叶8例，右上肺叶9例，右中肺叶1例，左下肺叶7例，左上肺叶11例，术后病理鳞癌13例，腺癌21例，大细胞癌2例。两组患者术前临床诊断均为可行肺叶切除的周围型病灶，肿瘤直径≤3 cm。两组患者术后病理分期均为Ⅰ期，术前均经过两倍剂量增强头颅核磁（magnetic resonance imaging, MRI）、发射型计算机断层（emission computed tomography, ECT）骨扫描和上腹部增强计算机体层摄影（computed tomography, CT）排除远处转移。术前检查提示患者可耐受心肺切除术近期无感染、无高血压、冠心病、糖尿病及自身免疫系统疾病史，无激素及影响免疫功能类药物服用史。两组肺癌患者的年龄、性别、病理分型及分期方面差异均无统计学意义（*P* > 0.05），具有可比性。

### 1.2 手术方法

胸腔镜组均采用胸腔镜三孔术式手术，全麻后患者于腋前线第7肋间做1 cm切口作观察孔，置入胸腔镜进行胸腔探查，于腋前线第3肋间或第4肋间做4 cm左右切口作主操作口，于腋后线第9肋间做1 cm切口作副操作孔。切口完成后，主操作口置入切口保护套，腔镜模式下进行肺叶切除，系统淋巴结清扫，安放引流管两根。开胸手术组采用传统的后外侧切口，探查病灶，进行标准的肺叶切除，系统淋巴结清扫及安放引流管两根。

### 1.3 观察指标

记录两组患者手术时间、术中出血量、淋巴结清扫个数、术后胸腔引流量、胸管留置时间。所有患者于术前1 d、术后3 d和7 d分别抽取外周静脉血，乙二胺四乙酸（ethylene diamine tetraacetic acid, EDTA）抗凝，取血后30 min内离心1, 000 *g*、15 min，于-20 ℃冰冻保存。采用酶联免疫法测定CRP、IL-6、IL-10和TNF-α水平。

### 1.4 统计学方法

选用SPSS 17.0统计软件，计量资料以均数±标准差（Mean±SD）表示，组间比较采用*t*检验；计数资料比较采用*χ*^2^检验，以*P* < 0.05为差异有统计学意义。

## 2 结果

### 2.1 手术相关指标的比较

胸腔镜组和开胸手术组的手术时间、术中出血量、淋巴结清扫个数、术后胸腔引流量、胸管留置时间均无明显差异（*P* > 0.05）。具体见表 1。

**1:** 胸腔镜组和开胸手术组患者手术相关临床资料 Surgery-related clinical data between VATS group and thoracotomy group

	VATS group	Thoracotomy group	*t*	*P*
Operation time (min)	142.72±11.70	146.85±10.69	0.496	> 0.05
Blood loss (mL)	112.64±39.54	116.82±42.70	0.247	> 0.05
Number of lymph node dissection	18.24±1.57	18.70±1.42	0.301	> 0.05
Chest drainage (mL)	580.22±72.41	597.26±80.70	0.533	> 0.05
Chest tube indwelling time (d)	3.68±0.72	3.72±0.81	0.467	> 0.05
VATS: video assited thoracoscopic surgery.				

### 2.2 手术前后炎性因子指标检测的比较

两组患者间术前1 d的CRP、IL-6、IL-10和TNF-α血清浓度无明显差异（*P* > 0.05）。两组患者术后3 d和7 d的CRP、IL-6、IL-10和TNF-α血清浓度较术前均有明显升高（*P* < 0.05），胸腔镜组血清浓度各个时间节点均低于开胸手术组（*P* < 0.05），见表 2-表 5。

**2:** 胸腔镜组和开胸手术组患者血清CRP变化水平比较 The level of serum CRP between VATS group and thoracotomy group

Time	VATS group	Thoracotomy group	*t*	*P*
1-day before operation	8.36±0.68	8.23±0.81	0.562	> 0.05
3-day postoperation	11.01±0.98	14.01±1.13	3.672	< 0.05
7-day postoperation	9.21±1.10	11.32±1.05	4.763	< 0.05
CRP: C-reactive protein				

**3:** 胸腔镜组和开胸手术组患者血清IL-6变化水平比较 The level of serum IL-6 between VATS group and thoracotomy group

Time	VATS group	Thoracotomy group	*t*	*P*
1-day before operation	135.63±14.37	136.01±13.93	5.267	> 0.05
3-day postoperation	145.85±13.75	156.36±14.03	3.856	< 0.05
7-day postoperation	136.11±13.21	149.31±12.96	2.763	< 0.05
IL: interleukin.				

**4:** 胸腔镜组和开胸手术组患者血清IL-10变化水平比较 The level of serum IL-10 between VATS group and thoracotomy group

Time	VATS group	Thoracotomy group	*t*	*P*
1-day before operation	259.78±21.74	158.56±22.23	3.857	> 0.05
3-day postoperation	281.45±17.78	307.65±18.43	4.261	< 0.05
7-day postoperation	262.47±21.56	279.63±22.41	4.917	< 0.05

**5:** 胸腔镜组和开胸手术组患者血清TNF-α变化水平比较 The level of serum TNF-α between VATS group and thoracotomy group

Time	VATS group	Thoracotomy group	*t*	*P*
1-day before operation	78.52±11.75	79.10±12.33	3.846	> 0.05
3-day postoperation	99.31±12.68	127.73±12.47	5.014	< 0.05
7-day postoperation	79.42±13.01	110.54±12.53	4.726	< 0.05
TNF-*α*: tumor necrosis factor-*α*.				

## 3 讨论

电视胸腔镜完成肺叶切除术自1993年被报道以来，因为其微创创伤小的优势，在临床上应用日臻广泛。对于临床分期为早期的肺癌，在治疗上与传统开胸手术取得同样的预后也已得到大家的共识^[1, 2]^。被视为20世纪末胸外科革命性的突破。

肺叶切除术是早期肺癌效果最确定的一种治疗方式，但对人体也会造成创伤，影响患者恢复。因此，达到最佳治疗效果的同时付出最小的手术创伤代价是现代胸外科学的共同目标，也是发展的方向^[3]^。肺叶切除术可引起患者机体发生急性期炎症反应，可首先累及肺部，严重者可以发展成为全身炎症反应综合症。在这其中，发挥重要作用的是细胞因子^[4]^。胸腔镜肺叶切除能否较传统开胸手术减少患者机体细胞因子的释放，减轻术后急性期炎症反应使患者早期顺利恢复是肺肿瘤外科关注的热点。

细胞因子主要介导和调节炎性反应及免疫应答，按照细胞因子的功能和结构，IL类和TNF是目前研究较多的两类。多种IL与炎症反应密切相关，按照功能可以分为促炎细胞因子和抗炎细胞因子。前者包括IL-6，后者包括IL-10。在手术的急性应激反应阶段，与炎症相关的IL在血清的水平明显升高，升高程度与炎性反应呈正相关。IL-10等抗炎细胞因子在血清中的变化程度，也能够间接反映炎症反应的程度。TNF-α具有活化单核巨噬细胞，提高中性粒细胞吞噬能力，促进T淋巴细胞和B淋巴细胞的增殖，适量的TNF-α对机体的反应是有利的，但过度升高的TNF-α会引起炎症加剧、多器官功能损伤。CRP作用机制是促进免疫细胞吞噬病原体，使补体经典途径激活，溶解病原体。CRP是肝脏在应激状态下在TNF-α和IL-6等因子作用下合成的，CRP水平能够客观地反映手术应激反应对机体的损伤程度^[5]^。

本研究中我们对胸腔镜早期肺癌根治术患者临床资料和术后炎性因子的变化进行研究，两组患者的临床资料中，年龄、性别、手术时间、术中出血量、术后胸管留置时间和胸引量均无差异。选取炎症因子CRP、IL-6、IL-10和TNF-α作为监测指标，选取术前1 d、术后3 d和7 d作为时间节点，与常规开胸手术作为对比，就这两种不同的治疗方式后，患者对手术创伤所带来的炎性反应状态的影响。

本研究中两组患者术前1 d的CRP、IL-6、IL-10和TNF-α无明显差异，在术后3 d，两组患者CRP、IL-6、IL-10和TNF-α水平较术前均增高，说明了两种术式对机体都有损伤，存在一定程度的应激和炎症反应，这与手术诱导急性炎症反应的存在相吻合。胸腔镜组患者CRP、IL-6、IL-10和TNF-α水平在任意节点上低于开胸手术组，并且在术后7 d基本恢复到术前1天水平，说明胸腔镜手术对患者机体的损伤较轻，能够降低肺叶切除术后的炎症反应。我们在胸腔镜行早期肺癌根治术，虽然手术时间，术中出血及术后引流等指标与开胸术式差别不大，但手术创伤小，对周围组织器官影响小，术后应激和急性炎性反应轻，这些可以使患者机体免疫系统处于相对平衡状态。有研究^[6]^认为IL-6的水平是机体免疫抑制强弱的反应，并成正相关关系，术后较高的IL-6水平意味着患者免疫抑制程度高，容易出现复发和转移，进而影响预后。对本研究中的两组患者来说，应长期随访，进行验证。

很多研究^[7, 8]^已证实，胸腔镜手术和开胸手术在早期周围型肺癌的切除范围以及预后方面效果是相同的。肺癌的发病人群主要是老年人，随着年龄的增长，易发生转移^[9]^，对手术应激反应的生理储备及对手术的耐受性也随之降低。以最小的手术创伤达到最佳的治疗效果，提高术后患者的生活质量是现代医学发展的趋势。我们的研究认为，全胸腔镜下行早期肺癌根治术可以减轻手术创伤对机体的损伤，避免严重炎症反应，使患者达到快速恢复。
